# Tailoring crystallization kinetics for scalable and efficient large-area perovskite light-emitting diodes

**DOI:** 10.1126/sciadv.aef3336

**Published:** 2026-06-03

**Authors:** Sung-Doo Baek, Yuanhao Tang, Hyuntae Choi, Shang Jiang, Qixuan Hu, Yu-Ting Yang, Hanjun Yang, Wenzhan Xu, Kyu Yoon, Devansh Drolia, Limei Wang, Syed Joy, Su Hye Jeong, Gangsan Lee, Vivek Narsimhan, Kenneth R Graham, Jeffrey T. Miller, Jianguo Mei, Yoon Ho Lee, Letian Dou

**Affiliations:** ^1^ Davidson School of Chemical Engineering, Purdue University, West Lafayette, IN 47907, USA.; ^2^ Department of Chemistry, Emory University, Atlanta, GA 30322, USA.; ^3^ Department of Chemistry, Purdue University, West Lafayette, IN 47907, USA.; ^4^ Department of Chemistry, University of Kentucky, Lexington, KY 40506, USA.; ^5^ Department of Materials Science and Engineering, Sungshin Women’s University, Seoul 01133, Republic of Korea.; ^6^ Birck Nanotechnology Center, Purdue University, West Lafayette, IN 47907, USA.

## Abstract

Scalable fabrication of uniform perovskite films is a critical bottleneck for large-area perovskite light-emitting diodes (PeLEDs), hindered by coffee-ring formation and heterogeneous crystallization upon scaling. Here, we report a multimodal solvent-engineering strategy enabling highly uniform films via ambient blade coating combined with vacuum-assisted solvent evaporation. Incorporating *N*-methyl-2-pyrrolidone (NMP) and acetonitrile (ACN) into a dimethylformamide (DMF)–based system synergistically modulates evaporation dynamics: It suppresses macroscopic solute segregation through Marangoni flow–induced redistribution, while tuning precursor coordination to regulate nucleation/phase conversion and promote radiatively efficient film formation. Consequently, the ternary formulation yields films with improved uniformity, reduced trap densities, and enhanced radiative efficiency. Near-infrared (NIR) PeLEDs achieve peak external quantum efficiencies of 25.2% (10 mm^2^), 22.1% (60 mm^2^), and 19.0% (224 mm^2^). A functional vein-imaging prototype is also demonstrated using a 224-mm^2^ device, highlighting the impact for large-area optoelectronic applications.

## Teaser

Controlling fluid dynamics enables scalable manufacturing of high-efficiency large-area perovskite light-emitting diodes.

## INTRODUCTION

Halide perovskites have emerged as promising emitters for next-generation light-emitting diodes (LEDs), owing to their high color purity, defect-tolerant electronic structure, and compatibility with low-cost, solution-based fabrication (*1*–*3*). Recent studies on perovskite LEDs (PeLEDs) have demonstrated marked improvements in both device efficiency and stability across a broad spectral range (*4*–*10*). In particular, within the near-infrared (NIR) spectral region—where conventional organic LEDs suffer from fundamentally lower radiative efficiency due to increased nonradiative decay associated with small molecular bandgaps (*11*)—NIR PeLEDs have rapidly achieved external quantum efficiencies (EQEs) surpassing 30%, highlighting their potential as high-performance NIR light sources (*4*, *7*). Despite these advances, scaling PeLEDs beyond small active areas (<10 mm^2^) remains a critical bottleneck. Large-area perovskite films frequently suffer from severe coffee-ring effects, uncontrolled solvent removal, and nonuniform crystallization, all of which degrade optical uniformity and device performance (*12*–*14*). These issues severely impede the manufacturability of PeLEDs, especially in the context of modern display technologies that rely on large-area mother-glass substrates (*15*). Thus, scalable routes for fabricating uniform, thin perovskite emissive layers are essential for practical PeLED deployment.

To address this challenge, meniscus-guided coating techniques, such as blade coating and slot-die coating, have attracted attention as viable alternatives to spin coating owing to their compatibility with large-area substrates (*16*). However, applying these methods to PeLEDs presents a unique challenge due to the stringent requirement for layer thickness. For efficient charge transport and radiative recombination, PeLEDs typically require perovskite films that are only tens of nanometers thick (*17*). However, producing uniform films at the nanometer scale over large areas presents a fundamental challenge: Dilute precursor solutions crystallize only after substantial solvent evaporation, making macroscopic solvent flow the governing factor for final film morphology. While strategies such as air knife–assisted coating can mitigate some nonuniformities, they often suffer from irregular airflow, potential contamination, and limited compatibility with scalable manufacturing (*18*). This motivates the development of an alternative strategy capable of controlling solvent evaporation and crystallization uniformly across large substrates without relying on high-pressure airflow.

Here, we establish a multimodal kinetic framework combined with vacuum-assisted blade coating to produce highly uniform perovskite films over large areas. Using a tailored dimethylformamide (DMF)/*N*-methyl-2-pyrrolidone (NMP)/acetonitrile (ACN) formulation, we demonstrate that ACN acts as a dual-function modulator: It hydrodynamically drives Marangoni recirculation to suppress coffee-ring formation and thermodynamically triggers rapid supersaturation to unlock the kinetic trap imposed by NMP coordination. Leveraging this controlled crystallization pathway, we demonstrate NIR PeLEDs with peak EQEs of 25.2% (active area: 10 mm^2^), 22.1% (60 mm^2^), and 19.0% (224 mm^2^), along with uniform emission from devices up to 462 mm^2^. A 224-mm^2^ device enables a proof-of-concept NIR vein-imaging demonstration, underscoring the practical utility of our scalable emitter. This work establishes a generalizable solvent-engineering framework for producing ultra-uniform, large-area perovskite films suitable for LEDs, solar cells, photodetectors, and other scalable thin-film technologies.

## RESULTS

### Ternary-solvent systems for uniform film

We developed a scalable coating strategy for high-performance PeLEDs by combining blade coating with vacuum-assisted solvent evaporation, enabling the formation of highly uniform, thin perovskite films without the use of an air knife. To satisfy the stringent requirements for thickness control and macroscopic uniformity in PeLEDs, we designed a ternary-solvent precursor system tailored to optimize both crystallization kinetics and morphological homogeneity.

**Fig. 1.**
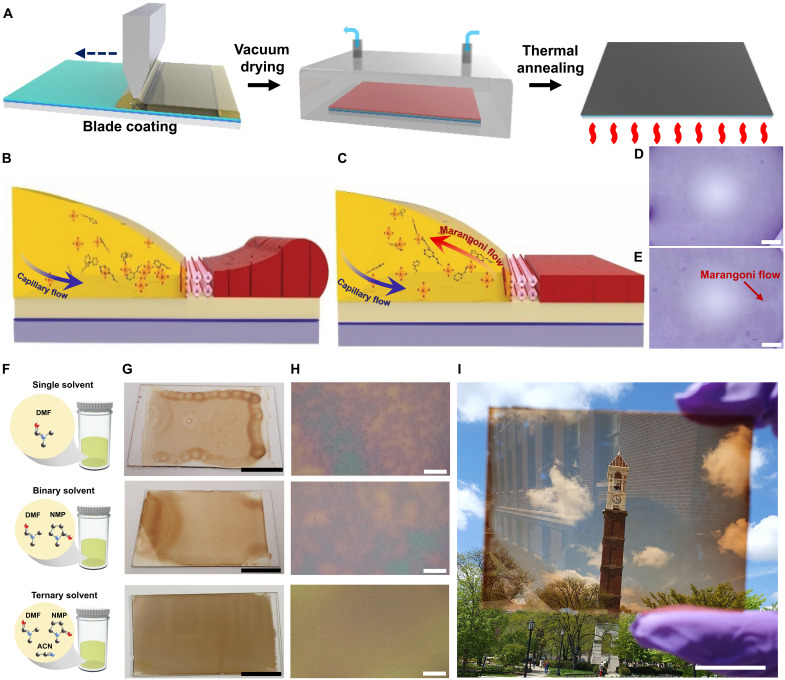
Suppression of macroscopic solute segregation via ACN-induced Marangoni recirculation. Schematic illustrations of (**A**) the vacuum-assisted blade-coating process for perovskite film fabrication, (**B**) the coffee-ring effect observed in perovskite precursor inks based on single- and binary-solvent systems, and (**C**) suppression of the coffee-ring effect in the ternary-solvent system through Marangoni flow–induced redistribution. OM images of solvent droplets containing PS microbeads dispersed in (**D**) the single-solvent system and (**E**) the ternary-solvent system, demonstrating the presence of Marangoni flow. Scale bars, 100 μm. (**F**) Schematic illustration of the single- (top), binary- (middle), and ternary-solvent (bottom) precursor solutions. (**G**) Photographs and (**H**) OM images of 75 mm–by–50 mm perovskite films prepared from single- (top), binary- (middle), and ternary-solvent (bottom) precursor solutions. Scale bars, 2 cm (G) and 100 μm (H). (**I**) Photograph of a 75 mm–by–50 mm perovskite film fabricated using the ternary-solvent precursor system, demonstrating macroscopic uniformity and a semitransparent appearance (clock tower visible through the film). Scale bar, 2 cm.

NMP was introduced into DMF to promote the crystallization of FAPbI_3_. The formation of PbI_2_-NMP intermediates is known to preserve coordination bonds and lower the formation energy of the α phase (*19*–*21*). To further regulate film formation during vacuum processing, we incorporated ACN, with a much higher vapor pressure than both DMF and NMP, as the third component (fig. S1). The perovskite precursor solution was blade-coated onto ZnO substrates and immediately transferred to a vacuum chamber for solvent removal, followed by thermal annealing (Fig. 1A). Unlike concentrated precursor inks used for thick solar cell films (*22*, *23*), PeLEDs require ~10-fold lower concentrations to produce emissive layers only tens of nanometers thick. At such dilute concentrations, film formation is delayed until a substantial fraction of the solvent has evaporated; consequently, vacuum-induced fluid dynamics become the dominant factor governing the macroscopic morphology (Fig. 1, B and C).

We investigated the droplet evaporation behavior and resulting film morphology across different solvent systems. In the single-solvent (DMF-only) system, evaporation is dominated by radially outward capillary flow, which transports solute to the droplet periphery. This results in a severe coffee-ring effect (Fig. 1B and fig. S2A), causing large thickness variations and morphological defects. The binary system (DMF/NMP) mitigated this effect to some extent but failed to eliminate edge accumulation (fig. S2B). In sharp contrast, the ternary system (DMF/NMP/ACN) induced strong Marangoni flow driven by surface tension gradients. This flow counteracts the outward capillary transport, promoting solute recirculation and effectively suppressing coffee-ring formation (Fig. 1C and fig. S2C). Optical microscopy (OM) using polystyrene (PS) microbeads as tracers confirmed that distinct Marangoni circulation occurs exclusively in the ternary system (Fig. 1, D and E).

Figure 1 (F to H) presents the schematic solvent compositions, macroscopic photographs, and corresponding OM images of perovskite films fabricated on 75 mm–by–50 mm glass/ZnO substrates (optimized ratios for binary and ternary systems are detailed in the following section). The single-solvent film exhibited numerous scattered coffee-ring patterns, whereas the binary film displayed pronounced solute accumulation at the edges. The ternary system, however, yielded highly uniform films with no observable ring deposition. OM analysis revealed a high density of cluster-like aggregates in the single-solvent film (Fig. 1H, top), which were partially reduced in the binary film and completely eliminated in the ternary film. Scanning electron microscopy (SEM) further indicated a slight increase in grain size for films processed with binary and ternary solvents compared to the single-solvent case (fig. S3). Figure 1I presents a photograph of the film fabricated using the ternary-solvent system on a large-area substrate. Background structures such as a clock tower and clouds are clearly visible through the film, demonstrating its high macroscopic uniformity. Thickness mapping (fig. S4) confirms that the ternary-solvent film maintains a consistent thickness of ~40 nm. In contrast, binary-solvent films suffered from severe edge thickening (>400 nm), while single-solvent films exhibited micrometer-scale ridges indicative of uncontrolled coffee-ring formation, which precluded accurate profilometry measurements.

### Photophysical properties of perovskite films

**Fig. 2.**
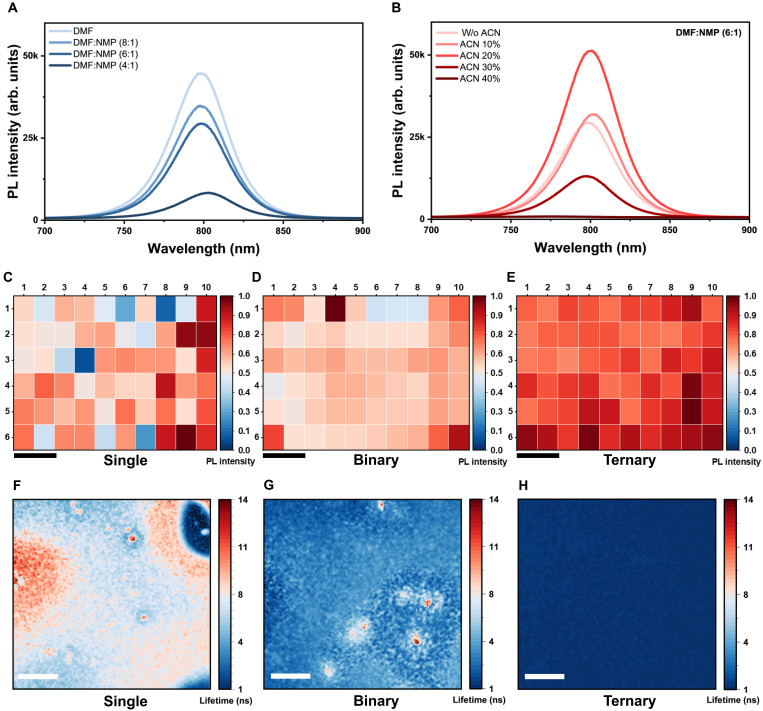
Optimization of optoelectronic quality and macroscopic homogeneity in ternary solvent–engineered perovskite films. Steady-state PL spectra of (**A**) single- and binary solvent–based perovskite films with different NMP volume ratios and (**B**) the ternary solvent–based perovskite films with varying ACN volume fractions (DMF:NMP fixed at 6:1). PL-intensity maps obtained from 60 measurement points over a 48 mm–by–28 mm region of the (**C**) single-, (**D**) binary-, and (**E**) ternary-solvent films. Scale bars, 1 cm. The single-solvent film displays severe macroscopic inhomogeneity, and the binary film shows reduced but still visible spatial variation, while the ternary film exhibits highly uniform emission across the entire mapped area. FLIM maps of the (**F**) single-, (**G**) binary-, and (**H**) ternary-solvent films reveal substantial lifetime fluctuations and defect-rich regions in the single- and binary-solvent films, whereas the ternary film shows a spatially uniform and short lifetime distribution indicative of suppressed nonradiative recombination. Scale bars, 20 μm.

To identify optimal processing conditions, we systematically varied the solvent ratios. In the binary system, we observed a clear trade-off between film morphology and emissive performance. Introducing NMP improved film uniformity relative to the DMF-only case (fig. S5); however, DMF:NMP = 8:1 still showed spatial nonuniformity, whereas further increasing NMP to 4:1 led to pronounced grain coarsening and mound-like features (figs. S5 and S6). Meanwhile, the photoluminescence (PL) intensity decreased monotonically with increasing NMP content (Fig. 2A), which we attribute to defect-assisted nonradiative recombination associated with residual NMP. We therefore selected DMF:NMP = 6:1 as the binary baseline, as it offers a meaningful improvement in uniformity without entering the severe coarsening regime at higher NMP fractions, while retaining sufficiently high PL for subsequent ternary optimization and mechanistic comparisons. Building on this optimized binary ratio, the ternary system demonstrated a synergistic enhancement in both morphology and performance. Unlike the binary system, the inclusion of ACN initially improved both film uniformity and optoelectronic properties. The PL intensity peaked at 20% ACN (Fig. 2B), coinciding with the formation of the most uniform and high-quality coating (figs. S7 and S8). However, beyond this point, excessive ACN (>30%) caused rapid aggregation and phase segregation, leading to a sharp drop in emission and the emergence of low-dimensional phases (fig. S9) (*24*). Consequently, the 20% ACN formulation was identified as the optimal condition, simultaneously maximizing film uniformity (fig. S10) and radiative efficiency.

While the steady-state PL spectra provide insight into the local optoelectronic quality, assessing the macroscopic homogeneity is crucial for large-area device performance. To quantify the spatial uniformity of emissive properties, we generated pixelwise-normalized PL-intensity heatmaps for large-area films prepared with single-, binary-, and ternary-solvent systems (Fig. 2, C to E). The single-solvent film exhibits a highly heterogeneous PL distribution characterized by a random mixture of low- and high-intensity regions. The binary-solvent film shows improved coverage compared to the single-solvent case but displays generally moderate and uneven PL intensity. In sharp contrast, the ternary-solvent film demonstrates a high-intensity and spatially uniform PL map, indicating high optoelectronic homogeneity across the film.

To further evaluate the spatial distribution of recombination dynamics, which complements the macroscopic PL-intensity mapping, we performed fluorescence lifetime imaging microscopy (FLIM) on the perovskite films prepared with different solvent systems (Fig. 2, F to H). The single-solvent film shows highly heterogeneous lifetimes, featuring large domains with prolonged lifetimes (>10 ns) adjacent to rapid-decay regions, indicative of severe local variations in film quality. The binary-solvent film exhibits improved coverage but still contains distinct localized hotspots representing phase or defect inhomogeneities.

In sharp contrast, the ternary-solvent film displays an exceptionally uniform lifetime distribution (~1 to 2 ns) across the entire mapped area. This uniformity indicates a spatially homogeneous recombination landscape with suppressed localized long-lived (defect-/trap-related) domains (*25*), in agreement with the macroscopic PL-intensity mapping. Subsequently, to quantify radiative efficiency, we focused our photoluminescence quantum yield (PLQY) evaluation on the optimized ternary-solvent film (fig. S11), as the severe macroscopic nonuniformity of single- and binary-solvent films precluded reliable measurements. At an excitation power density of 36.3 mW cm^−2^, the ternary-solvent film achieved a peak average PLQY of ~78% (with maximum values exceeding 80%), demonstrating highly efficient radiative recombination. Together, the high PLQY and the uniform FLIM lifetime suggest enhanced radiative recombination rather than increased nonradiative loss. Such a short (~1 to 2 ns) yet spatially homogeneous lifetime is favorable for LEDs, as it indicates rapid, uniform recombination with reduced trap-mediated losses.

### Mechanistic insights

**Fig. 3.**
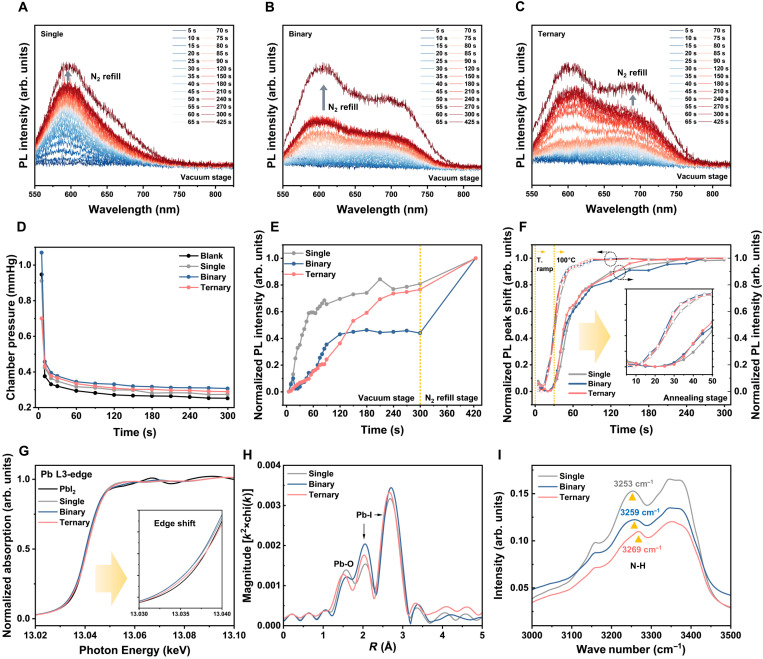
Unlocking kinetic traps and modulating precursor coordination for controlled crystallization. In situ PL spectra collected during the vacuum-pumping stage for perovskite films prepared using (**A**) single-, (**B**) binary-, and (**C**) ternary-solvent systems. (**D**) Real-time chamber-pressure evolution recorded simultaneously during the in situ PL measurement. (**E**) Corresponding normalized PL-intensity kinetics during the vacuum-pumping stage. (**F**) Normalized PL-intensity kinetics and PL peak shift during the subsequent thermal-annealing stage; the inset magnifies the early-stage PL evolution (5 to 50 s). T denotes the annealing temperature. (**G**) XANES spectra (the inset highlights the chemical shift of the Pb L3-edge), (**H**) EXAFS, and (**I**) FTIR spectra of the perovskite precursor solutions prepared with single-, binary-, and ternary-solvent systems.

To elucidate the influence of solvent engineering on nucleation dynamics, we monitored time-dependent in situ PL spectra during the vacuum-assisted blade coating (Fig. 3, A to C) and subsequent thermal-annealing stages (fig. S12). The spectral evolution reveals distinct crystallization pathways for the single-, binary-, and ternary-solvent systems. The DMF-only film exhibits a broad, singular emission peak that gradually intensifies (Fig. 3A). In contrast, both the binary and ternary systems display dual emissive features in the early stages, indicating the coexistence of distinct transient states: one associated with early DMF-induced nuclei and another associated with delayed NMP-stabilized intermediates (Fig. 3, B and C) (*19*–*21*). Notably, the binary film shows the most strongly suppressed PL evolution, whereas the ternary film demonstrates a steady signal increase.

To correlate these spectral behaviors with solvent evaporation kinetics, we analyzed the chamber pressure evolution (Fig. 3D) alongside the corresponding PL kinetics (Fig. 3E). The DMF-only film exhibits the most rapid pressure drop, driving immediate supersaturation and a steep initial PL rise; this uncontrolled rapid crystallization typically yields a broad crystallite size distribution. Conversely, the binary system shows the slowest pressure decay due to strong NMP retention, resulting in a prolonged low-intensity plateau followed by an abrupt PL surge only upon N_2_ refill. This confirms the delayed formation of NMP-stabilized intermediates. The ternary system, however, presents an intermediate yet continuous PL evolution with no abrupt transitions. This behavior suggests that the volatility of ACN accelerates evaporation while NMP moderates crystallization, establishing a regulated pathway that suppresses the coffee-ring effect and ensures spatial uniformity.

We further tracked the phase transformation from intermediate species to the emissive α phase during thermal annealing (Fig. 3F; full spectra in fig. S12). The evolution of PL peak position and intensity highlights critical differences in crystallization kinetics. In the initial stage (0 to 50 s), the single-solvent film exhibits the slowest redshift (Fig. 3F, open circles), indicating a slow transition from intermediate species to the α phase. In contrast, both binary and ternary films show faster peak shifts, reflecting the accelerated structural conversion facilitated by NMP. However, the PL intensity traces (Fig. 3F, solid circles) reveal a divergence in the establishment of efficient radiative pathways. Although the binary film undergoes a rapid structural phase transition (fast peak shift), its PL intensity rises slowly, reaching 95% of its maximum PL intensity only after ~210 s. This discrepancy suggests that while NMP accelerates phase formation, residual intermediates may retard the establishment of radiative pathways. The ternary system overcomes this limitation: It exhibits both a rapid phase transition and the fastest rise in PL intensity, achieving this benchmark by ~150 s. This result confirms that the addition of ACN facilitates the efficient removal of intermediates, thereby accelerating the onset of efficient radiative recombination and the formation of high-quality perovskite films.

To elucidate the molecular origins of the solvent-dependent crystallization behavior, we investigated the precursor coordination environment using synchrotron-based x-ray absorption near-edge structure (XANES) and extended x-ray absorption fine structure (EXAFS) (Fig. 3, G and H, and fig. S13). XANES measurements reveal a slight shift of the Pb L3-edge toward higher energy upon NMP/ACN addition, indicating a change in the local electron density around the Pb cation. Quantitative EXAFS fitting further confirms that while the Pb─I bond length remains negligibly changed, the iodide coordination number (CN) increases from 2.8 (single) to 3.1 (ternary) (Fig. 3H and table S1). This higher halide CN signifies a more fully coordinated Pb center, which effectively lowers the nucleation barrier. Crucially, the introduction of NMP elongates the Pb─O bond length from 2.28 Å (single) to 2.40 to 2.41 Å (binary and ternary). This structural elongation suggests that NMP forms more labile Pb-solvent complexes despite its high Lewis basicity (*26*, *27*). Such weakened Pb─O interactions facilitate bond cleavage during solvent evaporation, accelerating the transition of intermediates into the α phase, which is consistent with the rapid structural conversion observed in the in situ PL kinetics.

In parallel, liquid-phase Fourier transform infrared (FTIR) measurements (Fig. 3I) highlight the critical role of solvent-formamidinium iodide (FAI) interactions (*19*–*21*). Consistent with nuclear magnetic resonance (NMR) spectroscopy analysis revealing that only NMP undergoes substantial chemical shifts upon precursor interaction (fig. S14), the N─H stretching modes of FA exhibit a redshift upon NMP addition, signaling strengthened hydrogen bonding. Notably, the ternary system induces an even larger redshift; this effect is attributed to the modulation of the local solvation shell by ACN, which reinforces the hydrogen bonding between NMP and FA through partial displacement or steric confinement (*28*). Collectively, these results demonstrate that NMP and ACN synergistically reshape the coordination landscape—destabilizing Pb-solvent bonds while stabilizing FA intermediates—thereby accelerating nucleation kinetics and enabling the uniform crystallization observed in the ternary system.

### High-performance large-area NIR PeLEDs

**Fig. 4.**
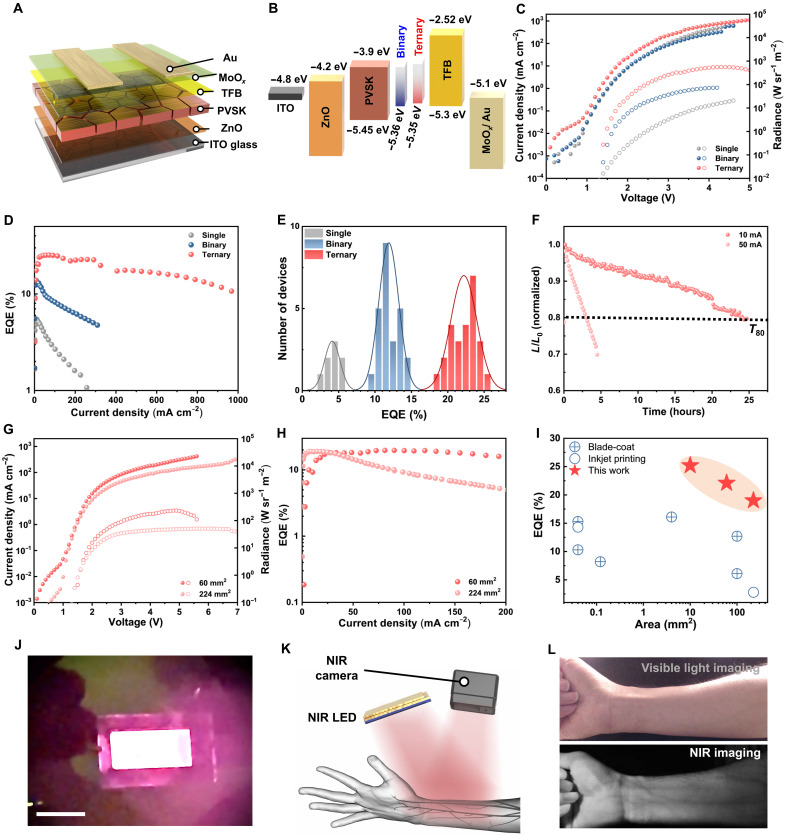
High-efficiency NIR PeLEDs with robust scalability and practical biomedical imaging utility. (**A**) Device architecture of NIR PeLEDs. (**B**) Energy band diagram of each functional layer determined from UPS measurements. (**C**) *J*-*V*-*R* characteristics (active area: 10 mm^2^), (**D**) EQE-*J* characteristics, and (**E**) peak EQE histograms of NIR PeLEDs fabricated with single- (*n* = 8), binary- (*n* = 25), and ternary-solvent (*n* = 25) systems. (**F**) Operational stability (*T*_80_) of the device fabricated using the ternary-solvent system measured at 10 and 50 mA cm^−2^. (**G**) *J*-*V*-*R* and (**H**) EQE-*J* characteristics of large-area PeLEDs with active areas of 60 and 224 mm^2^ fabricated with the ternary-solvent system. (**I**) Comparison of the EQE and device area of this work with previously reported large-area PeLEDs. Related references are provided in table S3 (*13*, *14*, *18*, *33*–*35*). (**J**) Photograph of 462-mm^2^ PeLED fabricated using the ternary-solvent system, showing uniform NIR emission across the entire area. Scale bar, 2 cm. (**K**) Schematic illustration of the NIR vein-imaging experiment. (**L**) Demonstration of vein imaging on a human wrist using the large-area NIR PeLED and an NIR-modified camera (visible-light image versus NIR image).

Leveraging our optimized large-area fabrication strategy, we constructed NIR PeLEDs with the following device architecture: indium tin oxide (ITO) (100 nm)/ZnO (25 nm)/perovskite/poly[9,9-dioctylfluorene-alt-*N*-(4-*sec*-butylphenyl)-diphenylamine] (TFB) (50 nm)/MoO*_x_* (10 nm)/Au (60 nm) (Fig. 4A). To elucidate the interfacial energy alignment, we performed ultraviolet photoelectron spectroscopy (UPS) (Fig. 4B and fig. S15). Notably, perovskite films fabricated with binary- and ternary-solvent systems exhibited shallower valence band maxima (VBMs) of 5.36 and 5.35 eV, respectively, compared to 5.45 eV in the single-solvent system. These shallower VBMs reduce the energy barrier for hole injection from TFB (~5.3 eV), facilitating more efficient charge transport, which may be attributed to the formation of a lower-dimensional phase at the perovskite surface (*24*, *29*), as evidenced by grazing-incident wide-angle x-ray scattering (GIWAXS) analysis (figs. S16 and S17).

The current density–voltage–radiance (*J*-*V*-*R*) characteristics of the devices are shown in Fig. 4C. The devices achieved maximum radiance values of 21, 73, and 555 W sr^−1^ m^−2^ for the single-, binary- and ternary-solvent systems, respectively, with the ternary system demonstrating superior radiance performance [electroluminescence (EL) peak at ~795 nm; fig. S18A]. This high radiance is consistent with the high device efficiency observed for the ternary system. As shown in Fig. 4D, the single- and binary-solvent devices exhibited limited peak EQEs of 5.5 and 13.8%, respectively, accompanied by substantial efficiency roll-off. In contrast, the ternary-solvent device achieved a much higher peak EQE of 25.2% (active area: 10 mm^2^) and maintained an EQE above 10% even at a high current density of 1000 mA cm^−2^. The peak EQE histograms further highlight the improved reproducibility of the ternary system (Fig. 4E): mean EQEs of 4.1, 11.9, and 22.2% with standard deviations of 1.06, 1.37, and 1.80% for the single-, binary-, and ternary-solvent systems, respectively. Notably, the coefficient of variation (SD/mean) decreases markedly from 25.9% (single) to 11.5% (binary) and 8.1% (ternary), confirming that the ternary system ensures the highest relative reproducibility alongside superior performance. Beyond efficiency, we evaluated the operational stability of the ternary-solvent device under different current densities (Fig. 4F). The ternary-solvent device exhibited *T*_80_ lifetimes of 23.4 hours at 10 mA cm^−2^ and 3 hours at 50 mA cm^−2^, indicating excellent operational stability under continuous driving (table S3).

To further assess the scalability of our fabrication strategy, we fabricated substantially larger NIR PeLEDs with active areas of 60 and 224 mm^2^ using the same ternary solvent–engineered perovskite films. As shown in Fig. 4 (G and H), both devices exhibit well-behaved diode characteristics and strong radiance output across the entire driving range. The 60-mm^2^ device achieves a high peak EQE of 22.1% with a maximum radiance of 232 W sr^−1^ m^−2^, while the 224-mm^2^ device maintains a still notable EQE of 19.0% and a radiance of 51 W sr^−1^ m^−2^, with both devices exhibiting similar EL peak positions (fig. S18B). Despite a nearly fourfold increase in device area, the efficiency loss remains minimal, demonstrating that the uniform crystallization enabled by the ternary-solvent system is preserved over large substrates. The slight efficiency drop in the large-area devices is primarily attributed to the series resistance of the ITO substrate, rather than the film quality. Further improvements can be achieved by minimizing these resistive losses through substrate engineering, such as incorporating metal grids or highly conductive transparent conducting oxides (*30*, *31*). Notably, the EQEs of our large-area NIR PeLEDs surpass those of previously reported large-area PeLEDs (Fig. 4I and table S3). To further validate the scalability of our coating approach, we also fabricated an even larger device with an active area of 462 mm^2^, which exhibited uniform NIR emission across the entire pixel area (Fig. 4J).

Last, to demonstrate the practical applicability of our large-area NIR PeLEDs, we integrated the 224-mm^2^ device into a vein-imaging setup (Fig. 4K). The PeLED served as a uniform NIR illumination source, with reflected light captured by a commercial NIR camera. As shown in Fig. 4L, veins that were difficult to discern under ambient visible light became resolved with high contrast under NIR imaging, owing to the strong and spatially uniform emission from the large-area perovskite source.

## DISCUSSION

In summary, we have established a scalable blade-coating strategy using a multimodal kinetic framework for dilute precursor inks, enabling the simultaneous achievement of macroscopic film uniformity, controlled crystallization, and superior optoelectronic quality across large areas. By synergizing vacuum-assisted evaporation with a tailored ternary formulation, we demonstrated that ACN acts as a dual-function modulator: inducing Marangoni recirculation to eliminate the coffee-ring effect, while accelerating supersaturation and regulating crystallization kinetics to enable uniform and radiatively efficient films. These advances enabled NIR PeLEDs with high efficiency, excellent operational stability, and strong scalability, from 10- to 224-mm^2^ and even 462-mm^2^ devices. Last, the successful demonstration of large-area NIR vein imaging underscores the practical utility of our approach, paving the way for biomedical visualization and next-generation large-area optoelectronic applications.

## MATERIALS AND METHODS

### Materials

The materials used in this study were FAI (99.99%, Greatcell Solar Materials), PbI_2_ (99.99%, TCI America), 5-aminovaleric acid (5AVA, 97%, Sigma-Aldrich), zinc acetate dihydrate (98%, Oakwood Chemical), tetramethylammonium hydroxide pentahydrate (98%, Thermo Fisher Scientific), TFB (*M*_w_ > 30,000, Lumtec), MoO_3_ (99.97%, Sigma-Aldrich), gold shots (99.999%, Kurt J. Lesker), DMF (anhydrous, ≥99.8%, Sigma-Aldrich), NMP (anhydrous, ≥99.5%, Sigma-Aldrich), toluene (99.9%, Sigma-Aldrich), ethanol (≥99.5%, Sigma-Aldrich), 2-propanol (IPA; ≥99.5%, Sigma-Aldrich), acetone (≥99.5%, Thermo Fisher Scientific), and Polybead PS spheres (1.0 μm microsphere, Polysciences Inc.).

### Preparation of precursor solution

The perovskite precursor solutions were prepared with FAI, PbI_2_, and 5AVA with a molar ratio of 2.5:1:0.7. They were first dissolved in DMF at 60°C for 30 min while stirring and then mixed with different solvent systems (NMP and ACN) right before fabricating films and devices. The molar concentration of PbI_2_ in the resulting solutions was maintained at 0.14 M. The ACN content (e.g., 20%) is defined as the volume percentage relative to the total precursor solution volume (v/v); the remaining solvent fraction follows the DMF:NMP ratio specified in the main text. ZnO nanocrystals were synthesized according to a previously reported method (*4*, *32*).

### Morphological characterizations

Film-thickness mapping was performed using a stylus profilometer (Bruker DektakXT). Thickness profiles of large-area perovskite films were obtained by scanning multiple positions across the substrate to evaluate macroscopic thickness uniformity. SEM images were obtained using a Hitachi S-4800 cold SEM microscope. Bright-field optical images were collected using a custom microscope (Olympus BX53). Photographs of the large-area perovskite films (75 mm by 50 mm) were captured using an iPhone 12 Pro under ambient lighting conditions.

### Steady-state optical measurements

The samples were excited with a light source [X-CITE 120Q ultraviolet (UV) lamp]. PL spectra were obtained using an Olympus BX53 microscope system equipped with spectrometer (SpectraPro HRS-300). PL-intensity mapping was performed by measuring PL signals at 60 spatially distributed points across each perovskite film fabricated on 48 mm–by–28 mm glass substrates. PLQY measurements were performed under ambient conditions using a custom-built setup equipped with a continuous-wave 375-nm laser (OBIS) as the excitation source, coupled with an integrating sphere and an optical fiber. Intensity calibration of the spectrometer was achieved using a National Institute of Standards and Technology (NIST)-traceable standard tungsten-halogen lamp (StellarNet SL1CAL). The perovskite films were deposited on ITO/ZnO substrates using the identical fabrication protocol used for the devices. To prevent degradation from oxygen and moisture, we spin-coated an inert encapsulation layer of isotactic poly(methyl methacrylate) (>80% isotactic, Sigma-Aldrich) from a 1 wt % anhydrous toluene solution at 4000 rpm for 30 s. To ensure statistical reliability, we derived PLQY values from 120 discrete points across the film immediately following fabrication.

### In situ PL kinetics

In situ PL measurements during the vacuum-assisted crystallization and thermal-annealing processes were carried out using an INSTEC heating and cooling stage (HCP421G-PM) equipped with a quartz observation window. The perovskite-coated substrates were placed inside the sealed stage, which was connected to an external vacuum line. A pressure sensor was installed inline between the chamber and the vacuum pump to monitor the real-time pressure evolution simultaneously with the PL acquisition. The same excitation source and detection system described in the “Steady-state optical measurements” section were used to continuously record the time-dependent PL spectra throughout the vacuum-pumping, N_2_-refill, and annealing steps.

### Grazing-incident wide-angle x-ray scattering

GIWAXS spectra were collected at beamline 7.3.3 of the Advanced Light Source at Lawrence Berkeley National Laboratory. These measurements were conducted with an incident angle from 0.1° to 0.9° and a wavelength of 1.24 Å (10 keV). Data integration was performed using Igor Pro 9.01 with the NIKA SAS 2D package.

### XANES measurements

Zn *K*-edge and Pb L3-edge XAS were conducted at the Brookhaven National Laboratory National Synchrotron Light Source II 8-ID Inner-Shell Spectroscopy beamline. The film samples were directly taped on the sample holder with Kapton tape, and the liquid samples were loaded into the PEEK liquid cell. The samples were scanned as received at their corresponding edge. The Zn and Pb foils were used to calibrate the edge energy to 9.659 and 13.035 keV, respectively. The XANES were normalized with linear and third-order polynomials in the pre-edge and post-edge regions, respectively. WinXAS was used to fit first shell peaks in the *k*^2^-weighted Fourier transform of the EXAFS data from *k* = 2.5 to 10.9 Å^−1^ for Zn and *k* = 2.7 to 10.0 Å^−1^ for Pb. FEFF6 was used to simulate phase and amplitude function for Zn─O/Pb─O scattering. Multiple scans of Zn and Pb samples were averaged to improve the data quality. The Debye-Waller factor (σ^2^) was obtained from the EXAFS fitting.

### FTIR and NMR measurements

Liquid-phase FTIR spectra were recorded using a Thermo Fisher Scientific Nicolet Nexus spectrometer. Proton NMR (^1^H NMR) spectra were obtained using a Bruker AV-III-400-HD spectrometer at 298 K. For NMR analysis, the precursor solutions prepared in their respective solvent systems were diluted with deuterated chloroform (CDCl_3_) prior to measurement.

### UPS measurements

UPS measurements were carried out using a PHI 5600 analysis system equipped with a hemispherical electron energy analyzer and a multichannel plate detector. The spectra were acquired using an H Lyman-α photon source (E-LUX 121) with a photon energy of 10.2 eV and a pass energy of 5.85 eV.

### Device fabrication

The ITO-coated glass substrates (109.2 mm by 27.2 mm for small area and 48 mm by 28 mm for large-area) underwent a sequential cleaning process in detergent (Alconox), deionized water, acetone, and IPA, each step involving sonication for 10 min. The cleaned ITO substrates were then treated with UV-ozone for 10 min. ZnO nanocrystals were subsequently spin-coated at 2000 rpm for 30 s and annealed at 150°C for 10 min. Following this, the substrates were transferred to a blade coater inside a dry box maintained at 20°C and 20% relative humidity (RH). A separate humidity-tolerance test was conducted at RH = 20, 30, and 40% (fig. S19). The perovskite precursor solutions were filtered through a 0.45-μm pore size polytetrafluoroethylene (PTFE) syringe filter and deposited by blade coating at a speed of 10 mm s^−1^ with a blade gap height of 50 μm. The substrates were then immediately transferred (within seconds) to a vacuum chamber connected to a N_2_-filled glove box and subjected to vacuum treatment (~1.3 kPa) for 5 min, to minimize uncontrolled ambient drying, followed by thermal annealing at 100°C for 16 min in the glove box. For small-area PeLEDs, ITO substrates with scribing lines were used, and the substrates were cut after annealing the perovskite film. For the hole transport layer, TFB dissolved in toluene (11 mg/ml) was deposited by spin-coating at 4000 rpm for 30 s. Last, a MoO*_x_* layer (10 nm) and a Au top electrode (60 nm) were deposited sequentially by thermal evaporation under high vacuum (<1 × 10^−6^ mbar). The active area of the devices was determined by the overlapping region of the ITO and Au electrodes, yielding device areas of 10, 60, and 224 mm^2^ for the data shown in this work.

### Device characterizations

The LED devices were characterized at room temperature within a N_2_-filled glove box without encapsulation. The *J*-*V* characteristics were recorded using a Keithley 2450 source-measure unit. Luminance characteristics, including radiance, EL, EQE, and operational stability, were obtained using a 100-mm PTFE integrating sphere connected to a spectrometer (Enli Technology, LQ-100X). The entire system was calibrated with a NIST-traceable standard QTH lamp. The operational stability of the LEDs was evaluated under constant current densities.

### NIR vein imaging

The large-area NIR PeLEDs (active area: 224 mm^2^) were encapsulated inside a N_2_-filled glove box using a thin glass cover and a two-part epoxy resin, followed by curing at room temperature for 1 hour. The encapsulated devices were operated under steady-state DC bias and positioned beneath the subject’s wrist at a similar height. Vein imaging was performed using a Logitech C920e webcam with the infrared-blocking filter removed. The camera was mounted above the illuminated region to collect reflected NIR light. Images were recorded under ambient lighting, converted to grayscale, and processed using contrast-limited adaptive histogram equalization to enhance local contrast and improve vein visibility.

## Supplementary Materials

### This PDF file includes:

Figs. S1 to S19 Tables S1 to S3 References
