# Supplementary material for: Tailoring crystallization kinetics for scalable and efficient large-area perovskite light-emitting diodes
Source: Sci Adv. 2026 Jun 3;12(23):eaef3336. doi: 10.1126/sciadv.aef3336 (PMC13232563; doi:10.1126/sciadv.aef3336)
Supplement: Supplementary file 1 — Figs. S1 to S19 Tables S1 to S3 References [file sciadv.aef3336_sm.pdf]

Science Advances
AAAS

Supplementary Materials for

Tailoring crystallization kinetics for scalable and efficient large-area perovskite light-emitting diodes

Sung-Doo Baek *et al.*

Corresponding author: Yoon Ho Lee, yoonhlee@sungshin.ac.kr; Letian Dou, letian.dou@emory.edu

Sci. Adv. **12**, eaef3336 (2026)
DOI: 10.1126/sciadv.aef3336

This PDF file includes:

Figs. S1 to S19
Tables S1 to S3
References

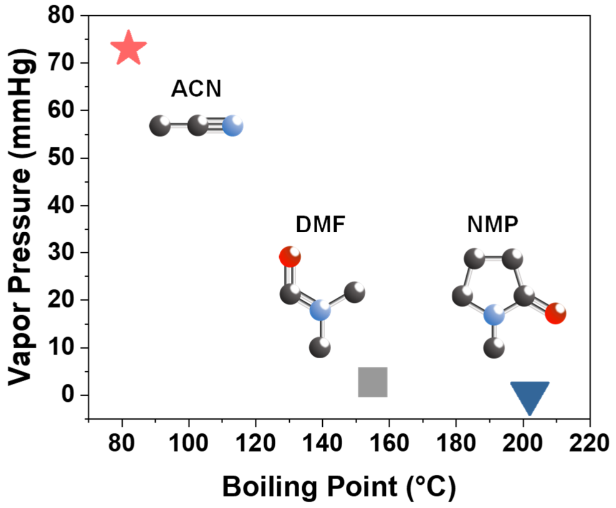

Fig. S1. Vapor pressures and boiling points of DMF, NMP, and ACN.

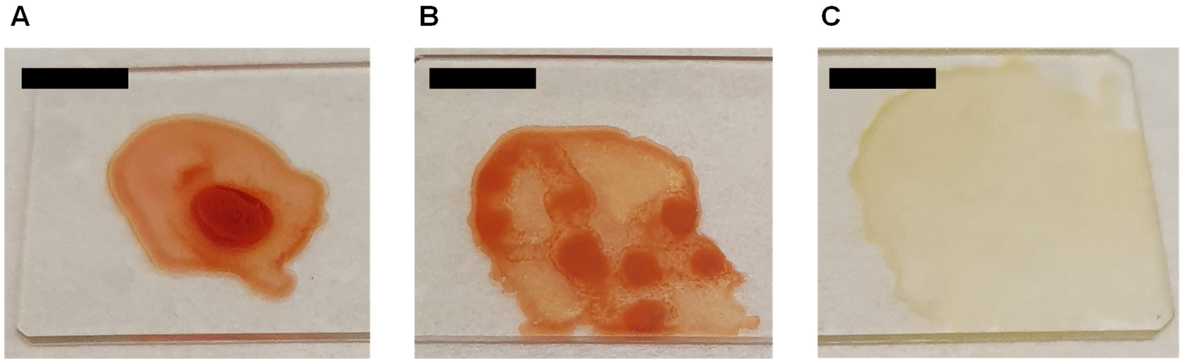

Fig. S2. Macroscopic appearance of drop-cast perovskite films from different solvent systems. Photographs of perovskite films prepared by drop-casting **(A)** single-, **(B)** binary-, and **(C)** ternary-solvent perovskite precursor solutions onto glass slides, followed by vacuum drying (scale bars: 1 cm).

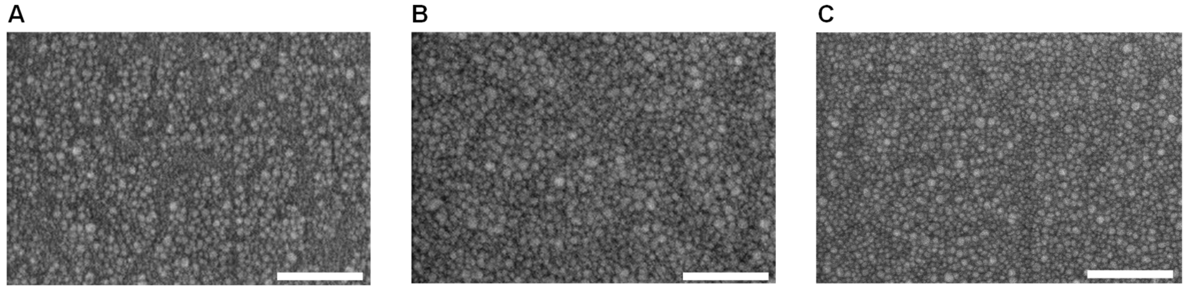

Fig. S3. Surface morphology of perovskite films prepared with different solvent systems.
SEM images of perovskite films prepared with **(A)** single-, **(B)** binary-, and **(C)** ternary-solvent systems (scale bars: 1 μm).

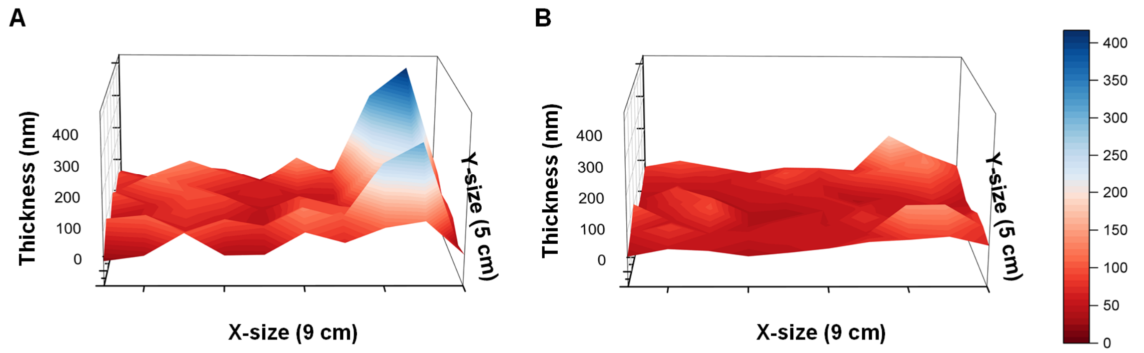

Fig. S4. Thickness uniformity across large-area films from binary- and ternary-solvent systems.

Thickness profiles measured across 90 × 50 mm^2 perovskite films prepared with **(A)** binary- and **(B)** ternary-solvent systems.

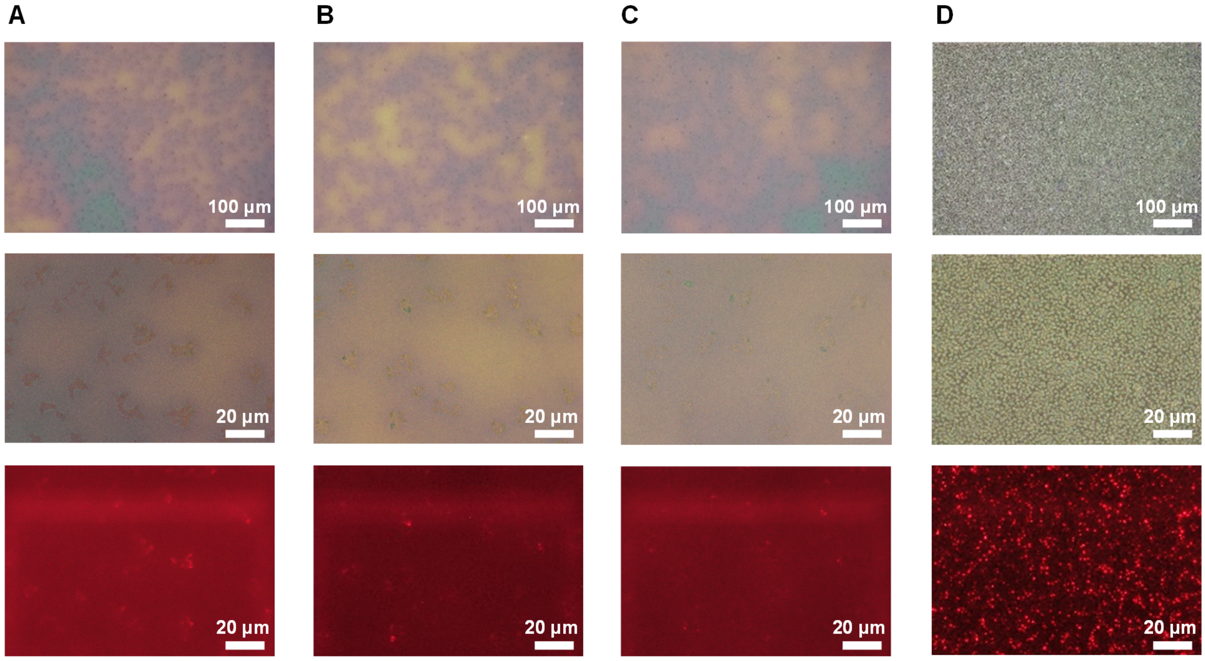

Fig. S5. Microscale morphology and emission homogeneity as a function of DMF:NMP volume ratio.

OM images (top), enlarged OM images (middle), and corresponding PL-OM images (bottom) of perovskite films prepared with **(A)** the single-solvent system (DMF-only) and the binary-solvent system (DMF/NMP) at DMF:NMP volume ratios of **(B)** 8:1, **(C)** 6:1, and **(D)** 4:1. The top-row OM images in panels (A) and (C) are reproduced from Fig. 1H top and middle panels for systematic comparison.

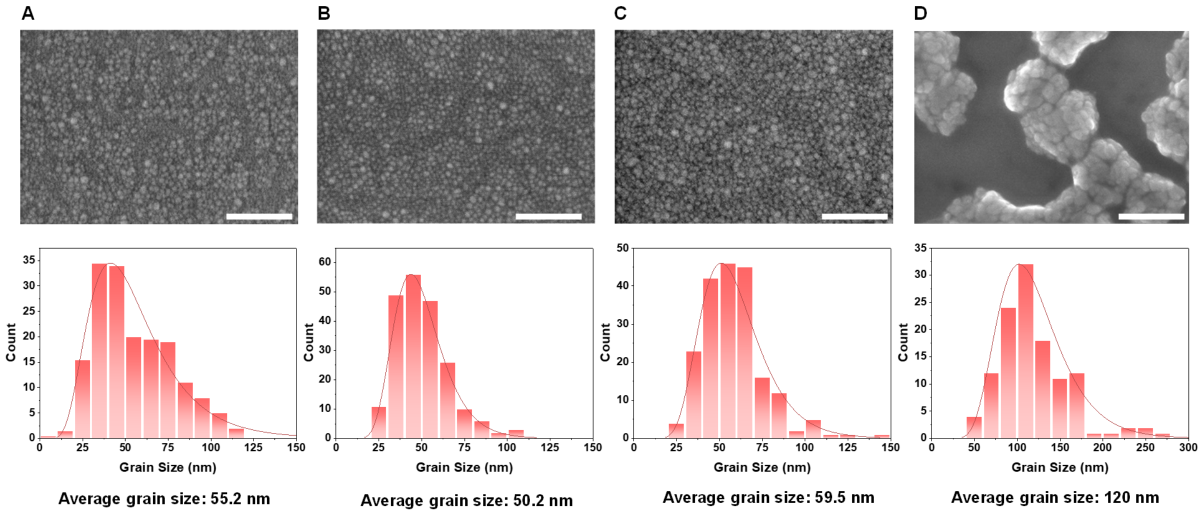

Fig. S6. Surface morphology and grain-size distribution as a function of DMF:NMP volume ratio.

SEM (top) and grain-size distributions (bottom) of perovskite films prepared wth **(A)** the single-solvent system (DMF-only) and the binary-solvent system (DMF/NMP) at DMF:NMP volume ratios of **(B)** 8:1, **(C)** 6:1, and **(D)** 4:1 (scale bars: 1 μm). The SEM images in panels (A) and (C) are reproduced from Fig. S3A and S3B respectively, for systematic comparison across DMF:NMP volume ratios alongside the corresponding grain-size distributions.

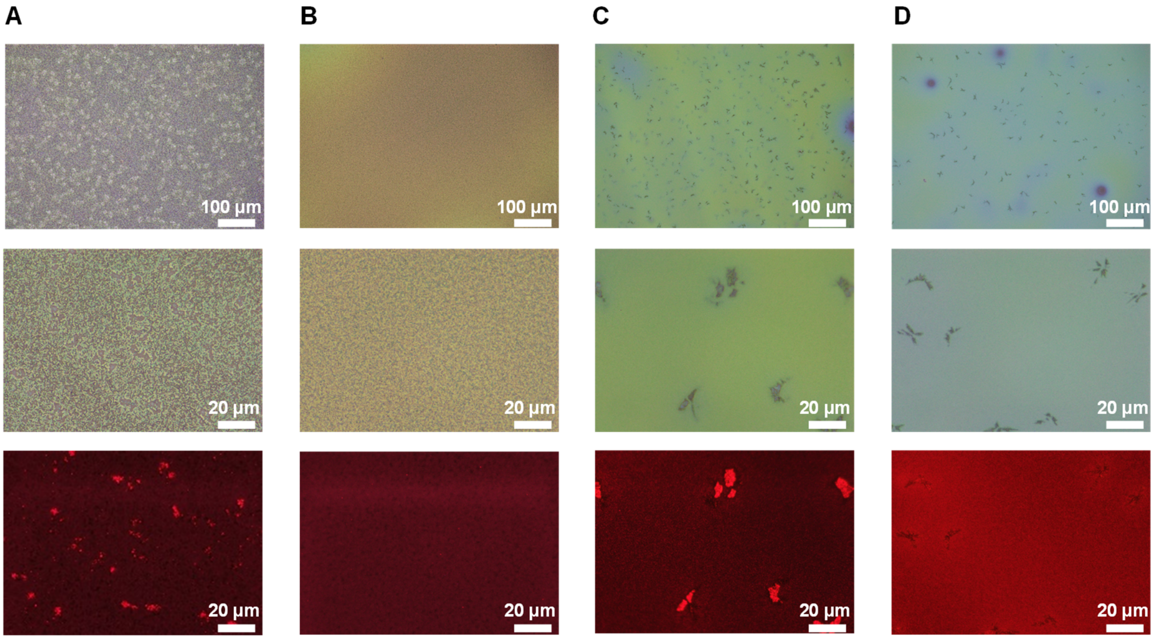

Fig. S7. Microscale morphology and emission homogeneity as a function of ACN volume fraction.

OM images (top), enlarged OM images (middle), and corresponding PL-OM images (bottom) of perovskite films prepared with the ternary-solvent system (DMF/NMP/ACN) at a fixed DMF:NMP volume ratio of 6:1 and ACN volume fractions of **(A)** 10%, **(B)** 20%, **(C)** 30%, and **(D)** 40%. The top-row OM image in panel (B) is reproduced from Fig. 1H bottom panel for systematic comparison.

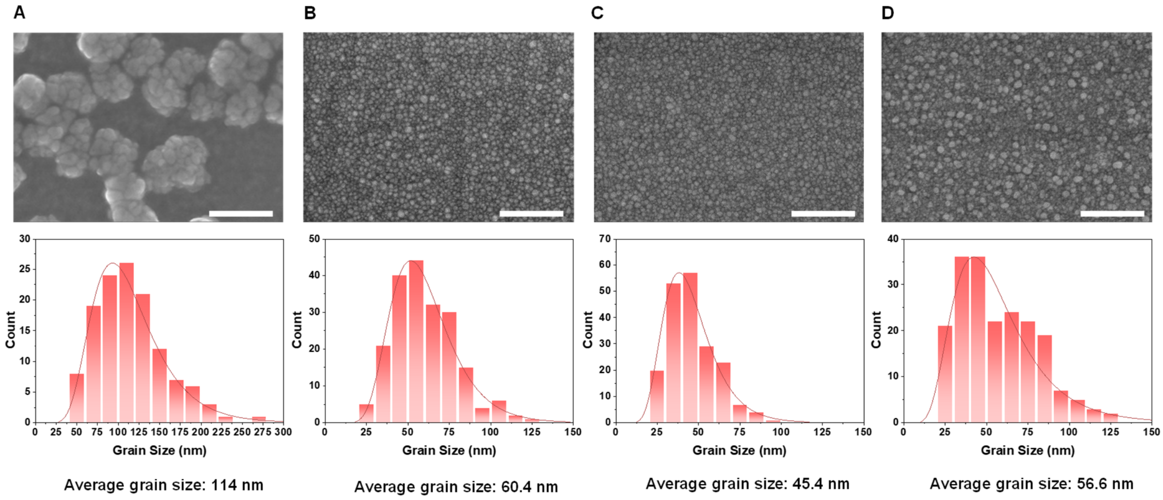

Fig. S8. Surface morphology and grain-size distribution as a function of ACN volume fraction.

SEM (top) and grain-size distributions (bottom) of perovskite films prepared with the ternary-solvent system (DMF/NMP/ACN) at a fixed DMF:NMP volume ratio of 6:1 and ACN volume fractions of **(A)** 10%, **(B)** 20%, **(C)** 30%, and **(D)** 40% (scale bars: 1 μm). The SEM image in panel (B) is reproduced from Fig. S3C for systematic comparison across ACN volume fraction alongside the corresponding grain-size distributions.

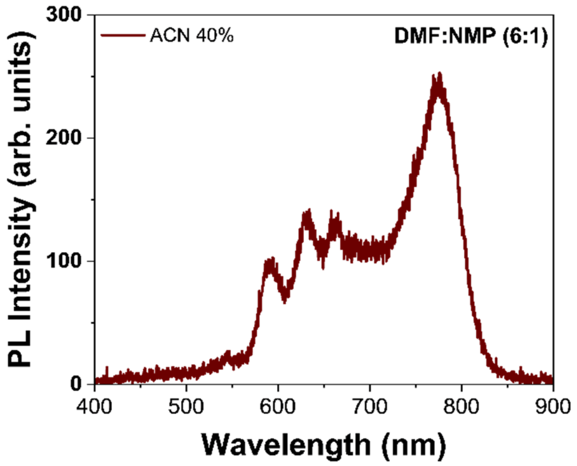

Fig. S9. Enlarged PL spectrum of a ternary-solvent film at 40% ACN volume fraction.

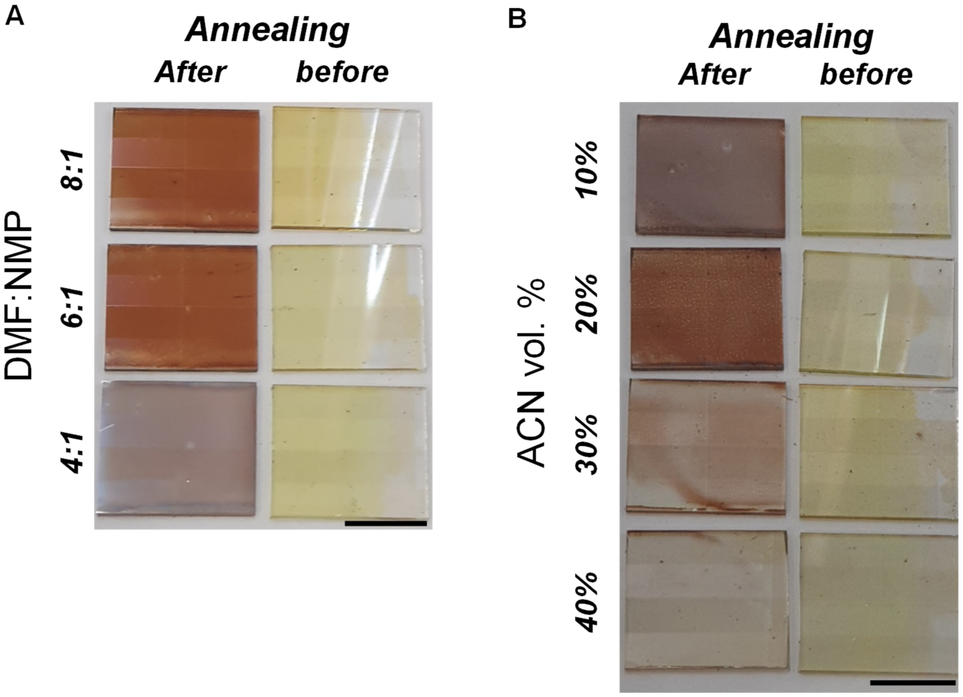

Fig. S10. Macroscopic film appearance before and after thermal annealing.
Photographs of perovskite films prepared using **(A)** the binary-solvent system and **(B)** the ternary-solvent system (DMF:NMP = 6:1), before and after thermal annealing (scale bars: 1.6 cm).

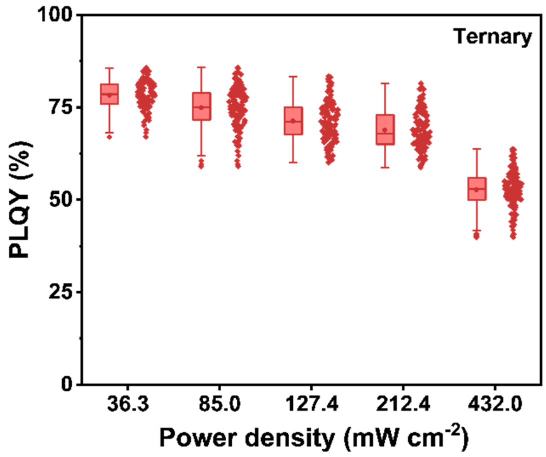

Fig. S11. Excitation-power-dependent PLQY distributions of the optimized ternary-solvent film.

Each distribution includes 120 measurement points per power level, displayed as box plots with overlaid data points.

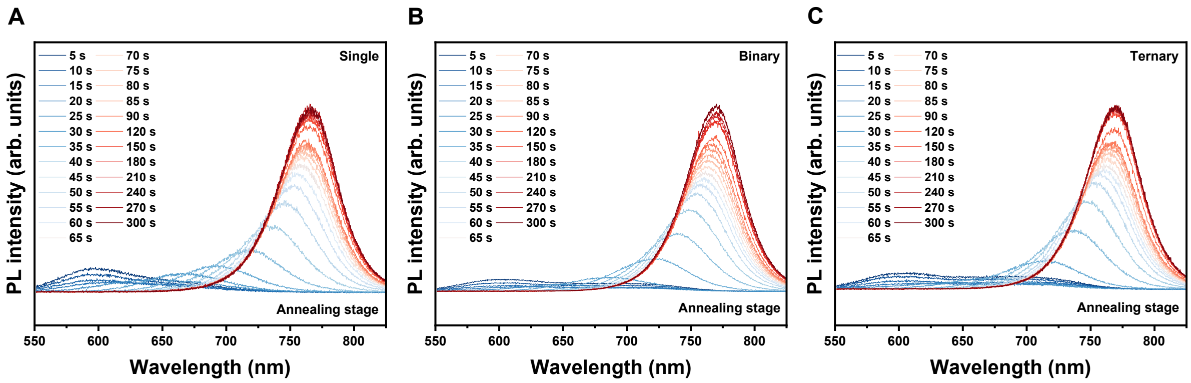

Fig. S12. In situ PL evolution during thermal annealing.
Spectra were continuously acquired at 100 °C for films prepared with **(A)** single-, **(B)** binary-, and **(C)** ternary-solvent systems.

Note: The PL peak blue-shifts to ~770 nm during thermal annealing due to the temperature dependence of the bandgap. At 100 °C, the bandgap increases relative to room temperature, resulting in emission at shorter wavelengths.

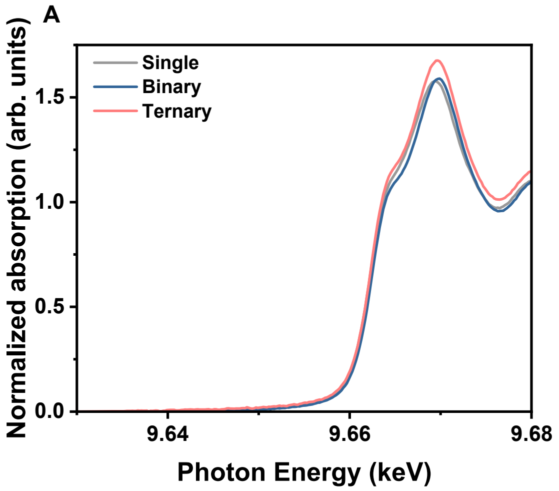

Fig. S13. Solvent-dependent local Pb coordination probed by Pb L3-edge X-ray absorption spectroscopy.

(A) XANES and **(B)** EXAFS spectra of perovskite films prepared with single-, binary-, and ternary-solvent systems on ZnO-coated substrates.

Note: Control measurements verified that the ZnO-coated substrate remains unaffected by the solvents, confirming that the observed spectral features originate exclusively from the perovskite precursors.

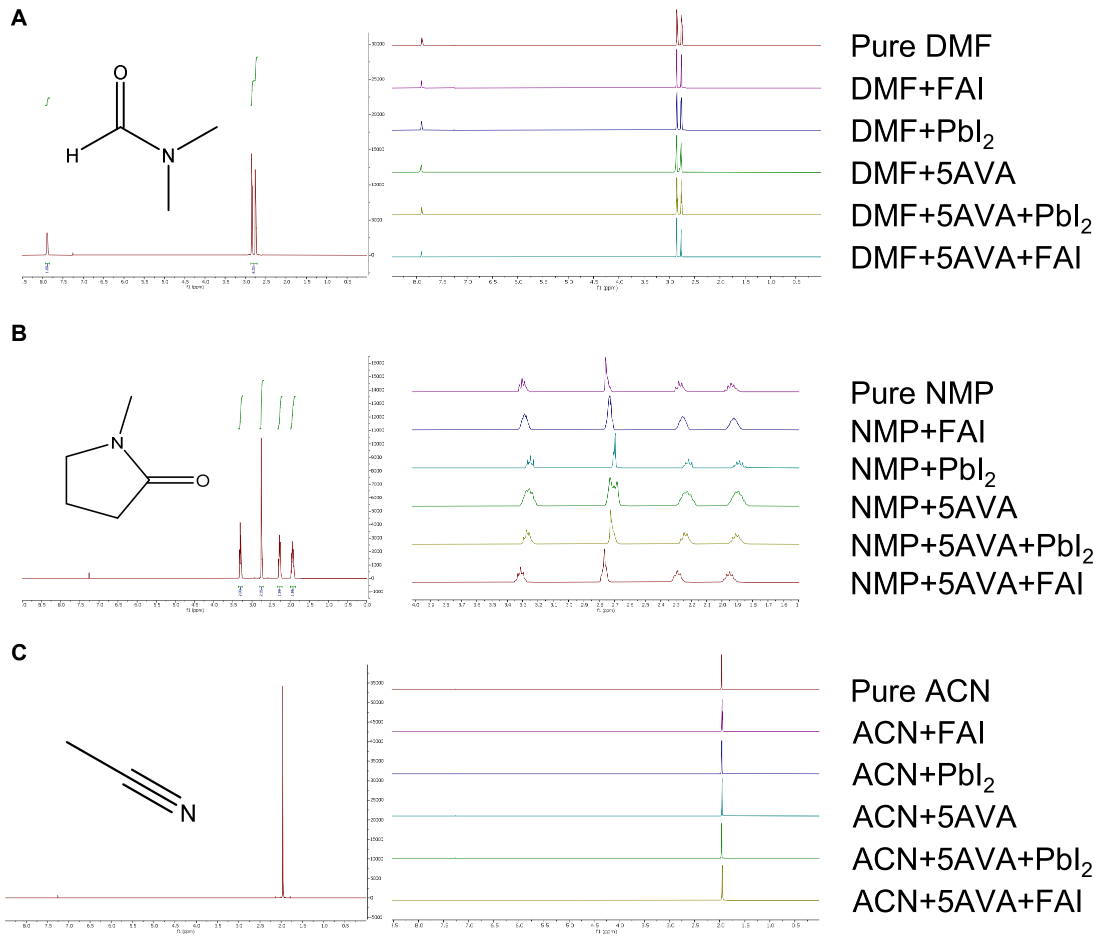

Fig. S14. Solvent-dependent ^{1}H-NMR chemical shifts of perovskite precursors. Spectra were acquired with PbI_2 and FAI dissolved in (A) DMF, (B) NMP, and (C) ACN.

Note: Consistent with its higher Kamlet–Taft β value (strong hydrogen-bond accepting ability) (*20*), NMP interacts more strongly with FAI than DMF. Accordingly, DMF and ACN show negligible changes in ^{1}H resonances upon addition of the precursor solutes, whereas NMP exhibits pronounced chemical shifts in the presence of PbI_2 and FAI, indicating strong coordination and hydrogen bonding.

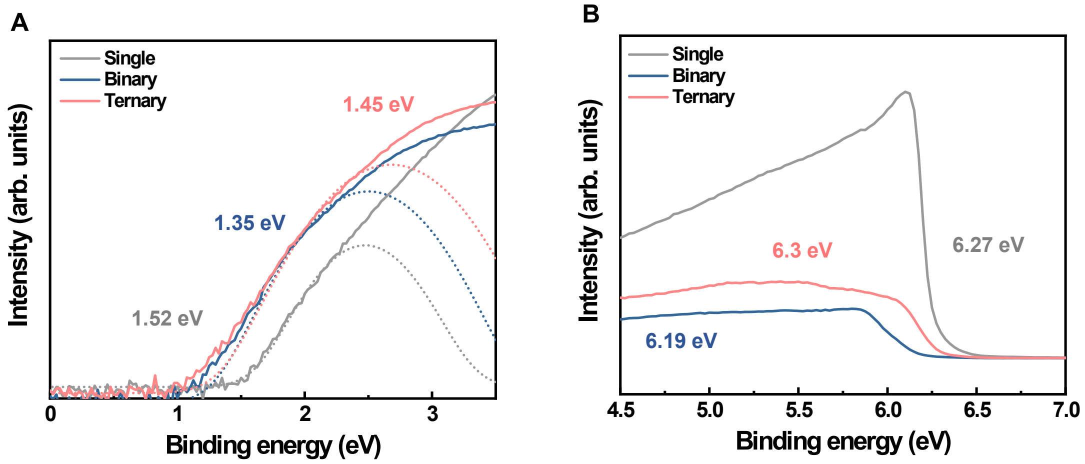

Fig. S15. UPS analysis of perovskite films prepared with different solvent systems.
(A) Secondary electron cutoff spectra and **(B)** valence-band spectra of perovskite films fabricated with single-, binary-, and ternary-solvent systems.

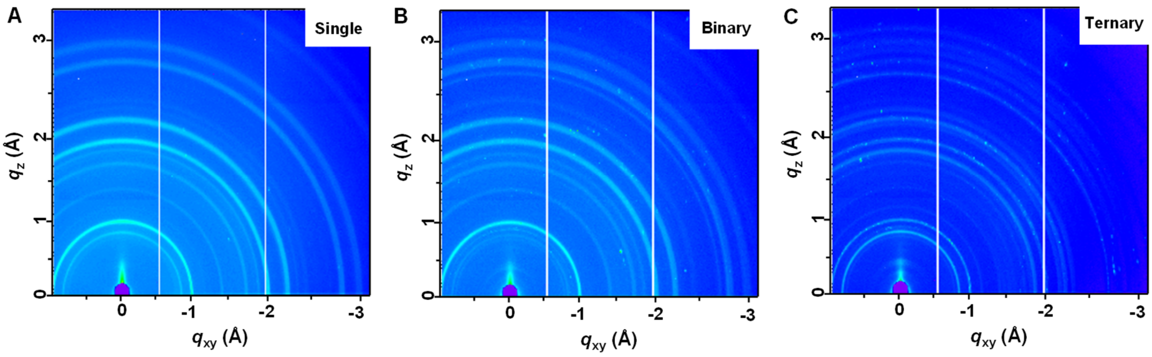

Fig. S16. Two-dimensional GIWAXS patterns of perovskite films from different solvent systems.

Films were prepared with **(A)** single-, **(B)** binary-, and **(C)** ternary-solvent systems.

Note: While the 3D α-$FAPbI_3$ phase constitutes the dominant crystalline framework across all solvent systems, additional diffraction features characteristic of low-dimensional phases emerge in the binary system and become more pronounced in the ternary-solvent system.

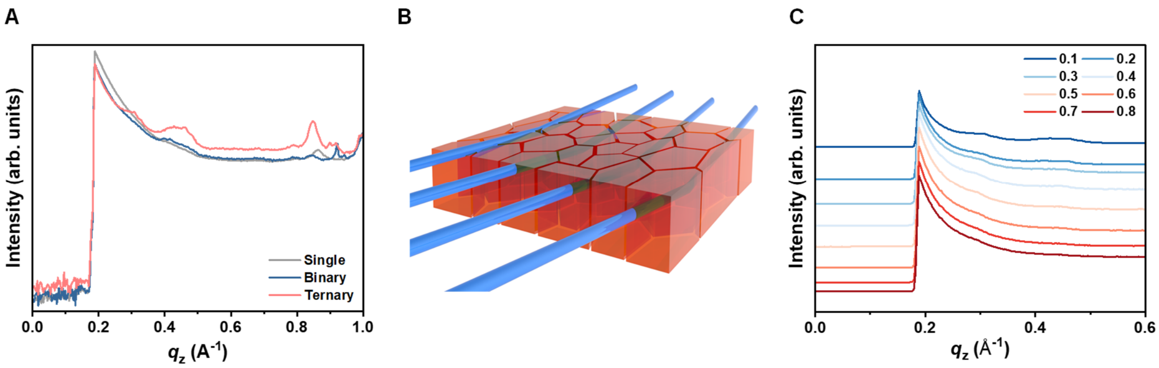

Fig. S17. Depth-resolved GIWAXS analysis via variable incidence-angle measurements. **(A)** Out-of-plane GIWAXS line profiles of perovskite films prepared with single-, binary-, and ternary-solvent systems. **(B)** Schematic of the incidence-angle geometry. **(C)** Angle-dependent line profiles of the ternary-solvent film.

Note: Low-dimensional phases are attributed to quasi-2D 5AVA-based perovskites. Angle-dependent GIWAXS analysis indicates that these phases are predominantly localized at the film surface.

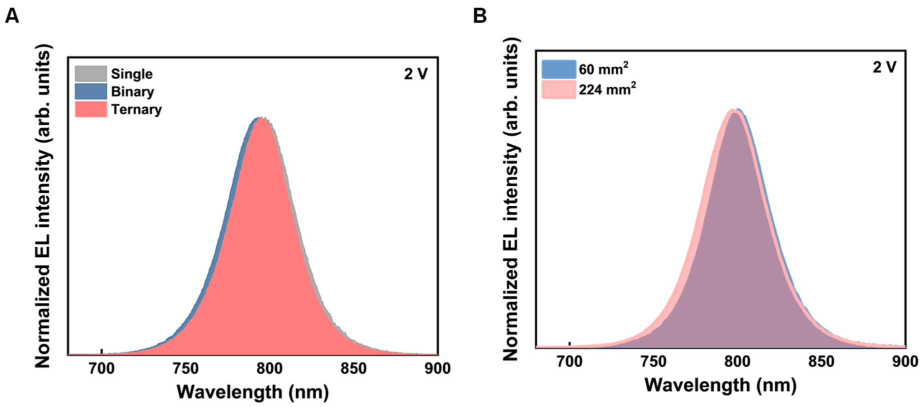

Fig. S18. EL spectra of PeLEDs with different solvent systems and active areas.
All spectra are normalized to peak intensity. **(A)** Devices with a 10 mm^2 active area fabricated using single-, binary-, and ternary-solvent systems. **(B)** Ternary-solvent devices scaled to active areas of 60 mm^2 and 224 mm^2.

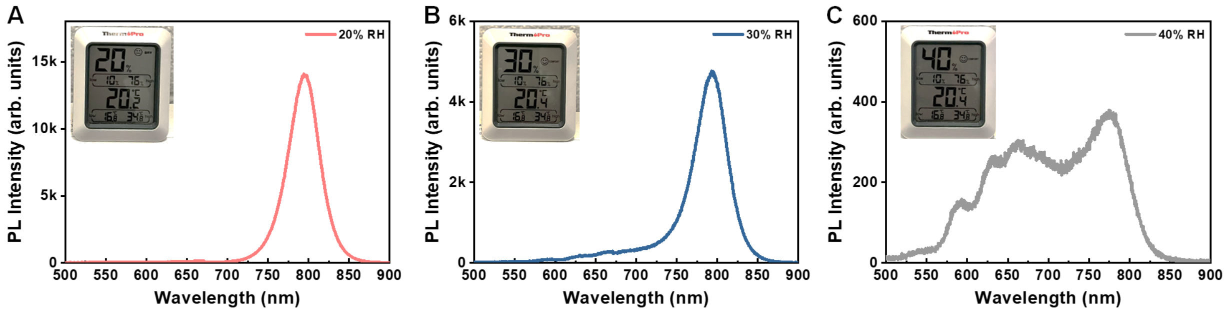

Fig. S19. Humidity dependence of blade-coated ternary-solvent films under ambient conditions.

Spectra were recorded on films blade-coated at **(A)** 20% RH, **(B)** 30% RH, and **(C)** 40% RH. Insets display photographs of the temperature and RH readings captured during deposition.

Note: Relative to 20% RH, films prepared at 30% RH show a marked decrease in NIR PL intensity and an additional emission feature in the 550–750 nm range. At 40% RH, the degradation becomes more severe. These trends suggest moisture-enhanced nonradiative losses and/or humidity-induced film heterogeneity during crystallization under ambient conditions.

Table S1. EXAFS fitting parameters for Pb L3-edge XAS of precursor solutions prepared with the single-, binary-, and ternary-solvent systems.

Pb L3-edge XAS						
Sample	Edge energy (keV)	Scattering path	CN	R (Å)	$\Delta\sigma^2$ (Å^2)	ΔE_0 (eV)
Single	13.033	Pb–I	2.8	2.96	0.013	–5.4
		Pb–O	0.3	2.28	0.005	–6.8
Binary	13.035	Pb–I	3	2.97	0.013	–3.3
		Pb–O	0.2	2.4	0.005	0.8
Ternary	13.0338	Pb–I	3.1	2.95	0.013	–6.3
		Pb–O	0.3	2.41	0.005	7.7

Table S2. EXAFS fitting parameters for Zn K-edge XAS of ZnO layers coated with perovskite films prepared with the single-, binary-, and ternary-solvent systems.

Zn K-edge XAS						
Sample	Edge energy (keV)	Scattering path	CN	R (Å)	$\Delta\sigma^2$ (Å^2)	ΔE_0 (eV)
Single	9.6621	Zn–O	3.9	1.96	0.004	0.94
Binary	9.6624	Zn–O	3.9	1.96	0.004	0.87
Ternary	9.6624	Zn–O	4	1.96	0.004	1.3

Table S3. Reported device characteristics of large-area PeLEDs.

EL peak (nm)	Fabrication method	Area (mm^2)	Peak EQE (%)	Operational stability (T_{80}; driving condition)	Device structure	Year	Ref. number
795	Blade coating	10	25.2	23.4 h at 10 mA cm^{-2}<br>3 h at 50 mA cm^{-2}	ITO/ZnO/$FAPbI_3$/TFB/MoO_x/Au	2026	This work
800		60	22.1	-			
799		224	19.0	-			
650		4	15.3	~16 s	ITO/PEDOT:PSS/Poly-TPD/$CsPbI_3$ QD/TPBi/Al	2024	33
489		4	10.3	~230 s at 100 cd m^{-2}	ITO/PVK/$CsPb(Br_{0.84}Cl_{0.16})_3$/TPBi/LiF/Al	2022	14
515		12	8.2	~2.6 min at 10 mA cm^{-2}	ITO/NiO_x/$(PEA)_2Cs_{n-1}Pb_nBr_{3n+1}$/TPBi/LiF/Al	2022	34
		100	6.1	-			
732		4	16.1	~9.3 h at 3 mA cm^{-2}	ITO/poly-TPD/$MAPbI_3$/TPBi/LiF/Al	2021	13
		100	12.7	-			
769	Inkjet printing	4	14.3	~1 h at10 mA cm^{-2}	ITO/PEDOT:PSS/Poly-TPD/$FA_{0.8}Cs_{0.2}PbI_3$/TPBi/LiF/Al	2024	18
520		225	2.8	~1 min at 90 cd m^{-2}	ITO/PEDOT:PSS/Poly-TPD/$FA_{0.3}Cs_{0.7}PbBr_3$/TPBi/LiF/Al	2020	35

REFERENCES

1. A. Fakharuddin, M. K. Gangishetty, M. Abdi-Jalebi, S. H. Chin, A. R. b. M. Yusoff, D. N. Congreve, W. Tress, F. Deschler, M. Vasilopoulou, H. J. Bolink, Perovskite light-emitting diodes. *Nat. Electron.* **5**, 203–216 (2022).

2. Y. Liu, Z. Ma, J. Zhang, Y. He, J. Dai, X. Li, Z. Shi, L. Manna, Light-emitting diodes based on metal halide perovskite and perovskite related nanocrystals. *Adv. Mater.* **37**, e2415606 (2025).

3. S. D. Baek, S. J. Yang, H. Yang, W. Shao, Y. T. Yang, L. Dou, Exciton dynamics in layered halide perovskite light-emitting diodes. *Adv. Mater.* **37**, e2411998 (2025).

4. S.-D. Baek, W. Shao, W. Feng, Y. Tang, Y. H. Lee, J. Loy, W. B. Gunnarsson, H. Yang, Y. Zhang, M. B. Faheem, P. I. Kaswekar, H. R. Atapattu, J. Qin, A. H. Coffey, J. Y. Park, S. J. Yang, Y.-T. Yang, C. Zhu, K. Wang, K. R. Graham, F. Gao, Q. Qiao, L. J. Guo, B. P. Rand, L. Dou, Grain engineering for efficient near-infrared perovskite light-emitting diodes. *Nat. Commun.* **15**, 10760 (2024).

5. J. Dong, B. Zhao, H. Ji, Z. Zang, L. Kong, C. Chu, D. Han, J. Wang, Y. Fu, Z.-H. Zhang, Y. Yang, L. Zhang, X. Yang, N. Wang, Multivalent-effect immobilization of reduced-dimensional perovskites for efficient and spectrally stable deep-blue light-emitting diodes. *Nat. Nanotechnol.* **20**, 507–514 (2025).

6. L. Kong, Y. Sun, B. Zhao, K. Ji, J. Feng, J. Dong, Y. Wang, Z. Liu, S. Maqbool, Y. Li, Y. Yang, L. Dai, W. Lee, C. Cho, S. D. Stranks, R. H. Friend, N. Wang, N. C. Greenham, X. Yang, Fabrication of red-emitting perovskite LEDs by stabilizing their octahedral structure. *Nature* **631**, 73–79 (2024).

7. M. Li, Y. Yang, Z. Kuang, C. Hao, S. Wang, F. Lu, Z. Liu, J. Liu, L. Zeng, Y. Cai, Y. Mao, J. Guo, H. Tian, G. Xing, Y. Cao, C. Ma, N. Wang, Q. Peng, L. Zhu, W. Huang, J. Wang, Acceleration of radiative recombination for efficient perovskite LEDs. *Nature* **630**, 631–635 (2024).

8. K. Wei, T. Zhou, Y. Jiang, C. Sun, Y. Liu, S. Li, S. Liu, X. Fu, C. Hu, S. Tian, Y. Yang, X. Fu, N. AlMasoud, S. M. H. Qaid, M. K. Nazeeruddin, H.-Y. Hsu, W.-D. Li, J. T. Kim, R. Long, W.

Zhang, J. Chen, M. Yuan, Perovskite heteroepitaxy for high-efficiency and stable pure-red LEDs. *Nature* **638**, 949–956 (2025).

9. J. S. Kim, J.-M. Heo, G.-S. Park, S.-J. Woo, C. Cho, H. J. Yun, D.-H. Kim, J. Park, S.-C. Lee, S.-H. Park, E. Yoon, N. C. Greenham, T.-W. Lee, Ultra-bright, efficient and stable perovskite light-emitting diodes. *Nature* **611**, 688–694 (2022).

10. X. Luo, W. Xu, G. Zheng, S. Tammireddy, Q. Wei, M. Karlsson, Z. Zhang, K. Ji, S. Kahmann, C. Yin, Y. Zou, Z. Zhang, H. Chen, L. A. B. Marçal, H. Zhao, D. Ma, D. Zhang, Y. Lu, M. Li, C. Deibel, S. D. Stranks, L. Duan, J. Wallentin, W. Huang, F. Gao, Effects of local compositional heterogeneity in mixed halide perovskites on blue electroluminescence. *Matter* **7**, 1054–1070 (2024).

11. A. Minotto, I. Bulut, A. G. Rapidis, G. Carnicella, M. Patrini, E. Lunedei, H. L. Anderson, F. Cacialli, Towards efficient near-infrared fluorescent organic light-emitting diodes. *Light Sci. Appl.* **10**, 18 (2021).

12. Y. Deng, Q. Wang, Y. Yuan, J. Huang, Vividly colorful hybrid perovskite solar cells by doctor-blade coating with perovskite photonic nanostructures. *Mater. Horiz.* **2**, 578–583 (2015).

13. S. Chu, W. Chen, Z. Fang, X. Xiao, Y. Liu, J. Chen, J. Huang, Z. Xiao, Large-area and efficient perovskite light-emitting diodes via low-temperature blade-coating. *Nat. Commun.* **12**, 147 (2021).

14. S. Chu, Y. Zhang, P. Xiao, W. Chen, R. Tang, Y. Shao, T. Chen, X. Zhang, F. Liu, Z. Xiao, Large-area and efficient sky-blue perovskite light-emitting diodes via blade-coating. *Adv. Mater.* **34**, e2108939 (2022).

15. R. Kaçar, R. B. Serin, E. Uçar, A. Ülkü, A review of high-end display technologies focusing on inkjet printed manufacturing. *Mater. Today Commun.* **35**, 105534 (2023).

16. R. Patidar, D. Burkitt, K. Hooper, D. Richards, T. Watson, Slot-die coating of perovskite solar cells: An overview. *Mater. Today Commun.* **22**, 100808 (2020).

17. M. Rai, L. H. Wong, L. Etgar, Effect of perovskite thickness on electroluminescence and solar cell conversion efficiency. *J. Phys. Chem. Lett.* **11**, 8189–8194 (2020).

18. H. Liu, G. Shi, R. Khan, S. Chu, Z. Huang, T. Shi, H. Sun, Y. Li, H. Zhou, P. Xiao, T. Chen, Z. Xiao, Large-area flexible perovskite light-emitting diodes enabled by inkjet printing. *Adv. Mater.* **36**, e2309921 (2024).

19. T. Bu, J. Li, H. Li, C. Tian, J. Su, G. Tong, L. K. Ono, C. Wang, Z. Lin, N. Chai, X. L. Zhang, J. Chang, J. Lu, J. Zhong, W. Huang, Y. Qi, Y. B. Cheng, F. Huang, Lead halide-templated crystallization of methylamine-free perovskite for efficient photovoltaic modules. *Science* **372**, 1327–1332 (2021).

20. X. Huang, G. Deng, S. Zhan, F. Cao, F. Cheng, J. Yin, J. Li, B. Wu, N. Zheng, Solvent gaming chemistry to control the quality of halide perovskite thin films for photovoltaics. *ACS Cent. Sci.* **8**, 1008–1016 (2022).

21. J.-W. Lee, Z. Dai, C. Lee, H. M. Lee, T.-H. Han, N. De Marco, O. Lin, C. S. Choi, B. Dunn, J. Koh, D. Di Carlo, J. H. Ko, H. D. Maynard, Y. Yang, Tuning molecular interactions for highly reproducible and efficient formamidinium perovskite solar cells via adduct approach. *J. Am. Chem. Soc.* **140**, 6317–6324 (2018).

22. X. Li, D. Bi, C. Yi, J. D. Décoppet, J. Luo, S. M. Zakeeruddin, A. Hagfeldt, M. Grätzel, A vacuum flash-assisted solution process for high-efficiency large-area perovskite solar cells. *Science* **353**, 58–62 (2016).

23. T. Wang, H. L. Loi, J. Cao, Z. Qin, Z. Guan, Y. Xu, H. Cheng, M. G. Li, C. S. Lee, X. Lu, F. Yan, High open circuit voltage over 1 V achieved in tin-based perovskite solar cells with a 2D/3D vertical heterojunction. *Adv. Sci.* **9**, e2200242 (2022).

24. Y. Shi, J. Hu, J. Chen, Y. Xu, W. Yang, J. Chen, Y. He, 5-Ammoniumvaleric acid stabilized mixed-dimensional perovskite submicron platelets with white light emission. *Nanoscale Adv.* **2**, 4822–4829 (2020).

25. Y. Yuan, G. Yan, C. Dreessen, T. Rudolph, M. Hülsbeck, B. Klingebiel, J. Ye, U. Rau, T. Kirchartz, Shallow defects and variable photoluminescence decay times up to 280 μs in triple-cation perovskites. *Nat. Mater.* **23**, 391–397 (2024).

26. S. Wintersteller, O. Yarema, D. Kumaar, F. M. Schenk, O. V. Safonova, P. M. Abdala, V. Wood, M. Yarema, Unravelling the amorphous structure and crystallization mechanism of GeTe phase change memory materials. *Nat. Commun.* **15**, 1011 (2024).

27. S. Roy, V. Bocharova, A. G. Stack, V. S. Bryantsev, Nucleation rate theory for coordination number: Elucidating water-mediated formation of a zigzag Na_2SO_4 morphology. *ACS Appl. Mater. Interfaces* **14**, 53213–53227 (2022).

28. X. Deng, Z. Cao, Y. Yuan, M. O. L. Chee, L. Xie, A. Wang, Y. Xiang, T. Li, P. Dong, L. Ding, F. Hao, Coordination modulated crystallization and defect passivation in high quality perovskite film for efficient solar cells. *Coord. Chem. Rev.* **420**, 213408 (2020).

29. M. Krummer, B. Zimmermann, P. Klingenberg, M. Daub, H. Hillebrecht, Perovskite-related 2D compounds in the system 5-amino valerian acid cation/MA/Pb/*X* (*X* = Cl, Br)—Synthesis, crystal structures, and optical properties. *Eur. J. Inorg. Chem.* **2020**, 4581–4592 (2020).

30. J. M. C. da Silva Filho, A. D. Gonçalves, F. C. Marques, J. N. de Freitas, A review on the development of metal grids for the upscaling of perovskite solar cells and modules. *Sol. RRL* **6**, 2100865 (2022).

31. D. H. Kim, J. B. Whitaker, Z. Li, M. F. A. M. van Hest, K. Zhu, Outlook and challenges of perovskite solar cells toward terawatt-scale photovoltaic module technology. *Joule* **2**, 1437–1451 (2018).

32. S.-D. Baek, D.-K. Kwon, Y. C. Kim, J.-M. Myoung, Violet light-emitting diodes based on *p*-CuI thin film/*n*-MgZnO quantum dot heterojunction. *ACS Appl. Mater. Interfaces* **12**, 6037–6047 (2020).

33. G. Shi, Z. Huang, R. Qiao, W. Chen, Z. Li, Y. Li, K. Mu, T. Si, Z. Xiao, Manipulating solvent fluidic dynamics for large-area perovskite film-formation and white light-emitting diodes. *Nat. Commun.* **15**, 1066 (2024).

34. C. Chen, L. Zeng, Z. Jiang, Z. Xu, Y. Chen, Z. Wang, S. Chen, B. Xu, Y. Mai, F. Guo, Vacuum-assisted preparation of high-quality quasi-2D perovskite thin films for large-area light-emitting diodes. *Adv. Funct. Mater.* **32**, 2107644 (2022).

35. D. Li, J. Wang, M. Li, G. Xie, B. Guo, L. Mu, H. Li, J. Wang, H.-L. Yip, J. Peng, Inkjet printing matrix perovskite quantum dot light-emitting devices. *Adv. Mater. Technol.* **5**, 2000099 (2020).
